# Opportunities and Limitations of Vascular Risk Factor Models in Studying Plasticity-Promoting and Restorative Ischemic Stroke Therapies

**DOI:** 10.1155/2019/9785476

**Published:** 2019-11-11

**Authors:** Dirk M. Hermann, Thorsten R. Doeppner, Aurel Popa-Wagner

**Affiliations:** ^1^Department of Neurology, University Hospital Essen, University of Duisburg-Essen, Essen, Germany; ^2^Center of Experimental and Clinical Medicine, University of Medicine and Pharmacy of Craiova, Craiova, Romania; ^3^Department of Neurology, University Medicine Goettingen, Goettingen, Germany

## Abstract

Major efforts are currently made promoting neuronal plasticity and brain remodeling in the postacute stroke phase. Experimental studies evaluating new stroke therapies are mostly performed in rodents, which compared to humans exhibit a short lifespan. These studies widely employ young, otherwise healthy, rodents that lack the vascular risk factors and comorbidities of stroke patients. These risk factors compromise postischemic neurological recovery and brain plasticity and in several contexts reduce the brain responsiveness to recovery-inducing plasticity-promoting treatments. By examining risk factor models, which have hitherto been used for studying experimentally induced ischemic stroke, this review outlines the possibilities and limitations of risk factor models in the evaluation of plasticity-promoting and restorative stroke treatments.

## 1. Introduction

Recent advances in recanalization therapies, i.e., the combination of thrombolytic drugs with interventional thrombectomy, have considerably increased clinical outcome in ischemic stroke patients [1]. Despite this progress, the large majority of ischemic stroke patients still exhibit neurological deficits in the long run, and ischemic stroke continues to be the most frequent cause of long-term disability. Neuroprotection therapies aiming at promoting the survival of previously ischemic tissue have failed in clinical trials. As a consequence, there has been a shift of focus from acute to postacute restorative therapies in the stroke field [2]. Indeed, major efforts are currently made to identify strategies allowing promoting neuronal plasticity and remodeling in the ischemic brain.

In view of the translation failure of neuroprotectants in clinical settings, the question arises if inadequate animal models may be responsible for the lack of action of new treatments in human stroke patients [3]. Animal studies are mostly performed in rodents, which compared to humans exhibit a short lifespan, a high ratio of grey to white matter, and a much smaller brain. These differences may result in inadequate conclusions, particularly when mechanisms of long-distance neuronal plasticity contributing to stroke recovery are analyzed. Compared with rodents, nonhuman primates apparently represent the far better stroke models. Due to ethical concerns, these models are rarely accessible.

Experimental stroke studies in rodents widely employ young, otherwise healthy, animals that lack the risk factors and comorbidities of stroke patients [2, 3]. Risk factors compromise neurological recovery. Studying risk factors is therefore important in the development of stroke therapies. Animal models of ischemic stroke have been reviewed by a number of papers in the past [3, 4], and in addition, aspects of risk factor modeling have more recently been evaluated by our group [5, 6]. By examining risk factor models, which have previously been used for studying experimentally induced ischemic stroke and stroke therapies, we now expanded previous works [5, 6], in which we evaluated stroke therapies from a more general perspective. The present review specifically highlights the possibilities and limitations of risk factor models in the evaluation of plasticity-promoting and restorative stroke therapies.

## 2. Insights from Hypercholesterolemia Models: Utility as Models of Cerebral Microangiopathy Resulting from Metabolic Syndrome, But Not as Cerebral Macroangiopathy Models

Hypercholesterolemia models used in ischemic stroke models have been established by targeted genetic mutations and/or high-cholesterol feeding in mice, rats, and rabbits.

The ApoE (apolipoprotein-E)^−/−^ mouse is the most widely used ischemic stroke model (Table 1). ApoE, which is expressed on chylomicrons, mediates reverse cholesterol transport to the liver. In comparison to wild-type controls, ApoE^−/−^ mice reveal ~4.3-8 times increased blood cholesterol concentrations [7, 8]. These concentrations are further elevated 1.4-2.7 times, when ApoE^−/−^ mice are kept on a high-cholesterol diet [7, 8]. Upon high-cholesterol diet, ApoE^−/−^ mice show fatty streaks in the aorta and extracranial carotid arteries after 6-10 weeks, which progress to atherosclerotic plaques after 6 months [7, 9]. Intracranial atherosclerosis is almost absent. In transient proximal or distal MCAO, infarct volume and neurological deficits were increased at 24-48 hours in ApoE^−/−^ mice on high-cholesterol diet [9, 10]. VEGF-induced angiogenesis was attenuated [11], and vasorelaxation was compromised, resulting in reduced cerebral blood flow upon MCAO [9, 11]. The exacerbation of infarct volume and neurological deficits involved excessive extracellular matrix breakdown and brain invasion of polymorphonuclear neutrophils [7, 8, 10]. Neutrophil depletion using anti-Ly6G antibody or neutrophil blockade using anti-CXCR-2 antibody prevented hypercholesterolemia-associated infarct volume exacerbation and neurological deficits [10].

Other models of hypercholesterolemia are the LDL receptor^−/−^ [12] and human ApoB- (apolipoprotein-B-) transgenic (hApoBTg) [13] mice (Table 1). LDL receptor controls cellular LDL and VLDL uptake by binding ApoB on LDL and VLDL. In humans, homozygous LDL receptor deficiency induces familial hypercholesterolemia that is characterized by more than 2.5 times increased blood cholesterol concentrations. When receiving regular diet, LDL receptor^−/−^ and hApoBTg mice show 1.9-2.4 times and 1.3-1.8 times elevated cholesterol concentrations, respectively [12, 13]. Blood cholesterol concentrations further increase 1.5 times and 2.2 times, respectively, on high-cholesterol diet [12, 13]. LDL receptor^−/−^ and hApoBTg mice reveal subtle vascular abnormalities when receiving normal diet [12, 13]. Upon high-cholesterol diet, the animals exhibit atherosclerotic plaques in the aorta and coronary arteries after 4-7 months [12, 13]. LDL receptor^−/−^ and hApoBTg mice on high-cholesterol diet reveal spontaneous cerebral microvascular occlusion and rarefication at ~6 months [14, 15]. Microvascular injury after unilateral common carotid artery occlusion was not influenced by transgenic ApoB [15], and infarct volume after transient proximal MCAO unaffected by LDL receptor^−/−^ [16]. Platelet deactivation by glycoprotein-Ib and glycoprotein-VI antibody reduced infarct volume and neurological deficits in 12-month-old LDL receptor^−/−^ and wild-type mice exposed to transient proximal MCAO without increasing hemorrhagic transformation [16]. Glycoprotein-IIb/IIIa inhibition had no effect.

ApoE^−/−^ mice have been crossbred with mice carrying LDL receptor^−/−^ [17], ATP-binding cassette transporter (ABCA1^−/−^) [18], endothelial nitric oxide synthase^−/−^ [19] or fibrillin-1 (C1039G) mutations [20], and LDL receptor^−/−^ mice with hApoBTg mice [21]. Some of these mice exhibit exacerbated atherosclerosis [17–19, 21] and others peculiar features, such as aneurysm formation [19] or intracranial atherosclerosis indicated by arterial wall thickening [21]. A particular feature of ApoE^−/−^ fibrillin-1 (C1039G)^+/-^ mice on high-cholesterol diet is atherosclerotic plaque rupture [20]. Unlike humans, mice and rats normally do not develop plaque rupture, which is a consequence of plaque vascularization and hemorrhage [20]. In humans, fibrillin-1 C1039G mutations are responsible for Marfan disease. The latter mice have not been studied in ischemic stroke models. ApoE^−/−^, LDL receptor^−/−^, and hApoBTg mice have strongly elevated blood cholesterol concentrations, which, if at all, are comparable to hereditary hypercholesterolemia. *In vitro*, LDL cholesterol dose-dependently reduces VEGF receptor-2 abundance on endothelial cells over a wide concentration range, resulting in the abrogation of VEGF-induced angiogenesis [22]. The clinical relevance of excessive blood cholesterol concentrations is vague. Care should be taken that potentially efficacious treatments are not prematurely abandoned by selecting risk factor conditions that hardly reflect conditions in humans.

When fed with a high-cholesterol diet, wild-type mice reveal 2.2-3.9 times elevated blood cholesterol concentrations (Table 1) [11, 13]. These mice exhibit only subtle fatty streaks in the aorta [13] but present a metabolic syndrome characterized by obesity, type-2 diabetes, and hypercholesterolemia [23] with lipid deposition in cerebral arterioles [7]. Despite elevated blood-brain barrier permeability and edema, neuronal injury and infarct volume after transient proximal MCAO were not altered in wild-type mice on high-cholesterol diet [7, 11]. This corresponds to human ApoE-*ε*3-transgenic (hApoE*ε*3Tg) and ApoE-*ε*4-transgenic (hApoE*ε*4Tg) mice exposed to permanent distal MCAO, in which infarct volume was also not elevated by high-cholesterol diet, although blood cholesterol concentration is increased 1.4 and 1.9 times, respectively [24]. Infarct volume is larger in hApoE*ε*4Tg than in hApoE*ε*3Tg mice on a regular diet [25], which has been attributed to direct neuroprotective effects of ApoE*ε*3.

In response to high-cholesterol diet, rats showed 2.1-4.2 times increased blood cholesterol concentrations (Table 1) [26, 27]. In transient proximal MCAO, infarct volume, blood-brain barrier permeability, brain edema, brain lipid peroxidation, and leukocyte infiltration were increased by high-cholesterol diet [26, 27]. Simvastatin and ginkgolide-B prevented these changes [26].

Upon high-cholesterol diet, rabbits exhibited 34.7 times elevated LDL concentrations (Table 1) [28]. After 5 weeks, atheromas were noted in the internal carotid and basilar arteries [28]. When exposed to focal cerebral ischemia induced by sephadex-G75 polymer injections, a high-cholesterol diet increased the mean infarct size probably as a consequence of platelet-rich thrombus accumulation, but reduced brain hemorrhage formation [28].

### 2.1. Lessons from Hypercholesterolemia Models

By promoting vascular lipid deposition in cerebral microvessels, hypercholesterolemia attenuates postischemic angiogenesis. Cerebral microangiopathy induced by high-cholesterol diet is similarly noted in wild-type and ApoE^−/−^ mice [7]. It is associated with disturbed cerebral hemodynamics [8, 9]. In view of the tight interactions of cerebral microvascular and brain parenchymal cells, cerebral microangiopathy compromises parenchymal brain remodeling and plasticity [7–11, 22, 26–28] and disturbs responses to restorative stroke treatments [9, 11, 22]. Importantly, rodents develop little intracranial large artery atherosclerosis, and atherosclerotic plaques lack plaque rupture, which as a precipitating event resulting in cerebral thromboembolism is responsible for the majority of ischemic stroke events. Wild-type mice with long-term exposure to high-cholesterol diet is a valuable model of metabolic syndrome [23], a frequent health problem in humans. This model is well suited for studying plasticity-promoting treatments.

## 3. Insights from Models of Arterial Hypertension: Utility as Models Unmasking Complication Risks and as Models of Premature Brain Ageing

Rat and mouse models of arterial hypertension were generated by inbreeding, targeted mutations, or pathophysiological stimuli. Although hypertensive strains exist in species other than rodents (e.g., spontaneously hypertensive rabbit), these strains have not been examined in ischemic stroke models.

Spontaneously hypertensive rats (SHR) were obtained by breeding Wistar-Kyoto rats (Table 2) [29]. Systolic blood pressure values of 180-200 mmHg are noted in these rats. SHR display hypertensive end-organ damage features including cardiac hypertrophy and renal insufficiency. Apart from impaired endothelial reactivity, gross vascular abnormalities, specifically atheromatosis and vascular thrombosis, are absent [30]. In permanent focal cerebral ischemia induced by transcranial MCAO or photothrombosis, the deactivation of the axonal growth inhibitor NogoA, which limits neural plasticity postinjury [31], by delivery of a NogoA-neutralizing antibody increased sensorimotor recovery and contralesional corticospinal plasticity after 12 weeks in SHR and normotensive rats, when delivered 24 hours poststroke [32]. In the brains of SHR, only subtle changes of neurotrophic factors and their receptors were found [33]. Postischemic neurogenesis and oligodendrogenesis were reduced in middle-aged (i.e., 12-month-old) compared to young (3-month-old) SHR with transient proximal MCAO [34], indicating premature brain ageing as a consequence of chronic hypertension.

By mating SHR with high blood pressure values, stroke-prone SHR (SHR-SP) were generated (Table 2). Their offspring reveal severe hypertension (systolic blood pressure > 200 mmHg) and spontaneous stroke at 9-13 months. Stroke development is accelerated upon salt-rich diet [35]. Neurological symptoms are observed at ~12 weeks. Prior to brain infarcts, cerebral blood flow decreases in the whole brain, reaching critical thresholds for neuronal injury [35]. Following unilateral common carotid artery occlusion, tissue oxygen is markedly decreased in SHR-SP white matter, resulting in extracellular matrix degradation, perivascular immune cell infiltrates, and myelin breakdown [36].

The Dahl salt-sensitive rat (DSR) was raised by breeding Sprague-Dawley rats exhibiting severe hypertension upon exposure to high-salt diet (Table 2). Gene sequencing revealed mutations in the angiotensin-converting enzyme and atrial natriuretic peptide receptor genes. Systolic blood pressure increases to 200-240 mmHg [37]. Cardiac hypertrophy and glomerular sclerosis are found, with cardiac failure at ~4-5 months. Up to 54% of DSR on high-salt diet developed fulminant spontaneous ischemic brain infarcts associated with massive aortic thickening, neurological deficits, and death [37]. Adenoviral kallikrein delivery prevented infarct development [37].

The mRenTg mouse expresses mouse renin under a liver-specific albumin promoter/enhancer, resulting in systolic blood pressure values of 160-180 mmHg at 3-8 months, cardiac hypertrophy, and proteinuria (Table 2). 50% of males died at 6-8 months. After transient proximal MCAO, infarct volume did not differ between mRenTg and wild-type mice [16]. Platelet deactivation by antiglycoprotein-Ib or antiglycoprotein-VI antibody reduced infarct volume in mRenTg and wild-type mice without increasing hemorrhagic transformation [16]. Glycoprotein-IIb/IIIa inhibition did not influence infarct volume, but increased brain hemorrhages more strongly in mRenTg than in wild-type mice [16].

The hRenAngTg mouse expresses human renin and human angiotensinogen flanked by their regulatory sequences, resulting in systolic blood pressure values of 160-180 mmHg (Table 2). Blood pressure in mice expressing human renin or human angiotensinogen is normal. When exposed to high-salt diet and treated with an endothelial NO synthase inhibitor, spontaneous brain hemorrhages appeared in hRenAngTg mice [38]. After permanent proximal MCAO, infarct volume and neurological deficits were increased in hRenAngTg mice compared to wild-type mice [39]. Effects were attributed to neuronal death-promoting actions of angiotensin-II. *Ex vivo*, neuronal injury after oxygen-glucose deprivation was more severe in brain slices obtained from hRenAngTg than from wild-type mice [39]. This effect was abolished by the angiotensin type-1 receptor inhibitor losartan [39].

Secondary renovascular hypertension in the rat has been modeled by renal artery clipping, by either leaving the contralateral kidney intact (“two kidneys, one clip”) or clipping both kidneys (“two kidneys, two clips”) (Table 2) [40, 41]. Systolic blood pressure values of 200-225 mmHg are noted [40]. Cardiac hypertrophy is found. After permanent proximal MCAO, “two kidneys, one clip” rats revealed increased infarct volume [41]. Five to twelve weeks after clipping, spontaneous cortical and subcortical infarcts, petechial hemorrhages, and brain atrophy were noted [40, 41]. These spontaneous strokes were not associated with overt neurological deficits.

### 3.1. Lessons from Arterial Hypertension Models

Arterial hypertension models are very suitable models for characterizing complication risks of stroke treatments, namely blood-brain barrier breakdown, brain edema, and intracerebral hemorrhage [16, 36]. Besides, they might be suitable for studying premature brain ageing [34]. In response to stroke, neuronal plasticity responses are apparently only modestly altered in hypertension models. An important aspect in the selection of hypertension models is associated comorbidities. Hypertensive end-organ damage, such as cardiac failure, compromises neurological recovery and animal survival [16, 37, 40, 41]. Models exhibiting end-organ damage are not ideal for evaluating restorative stroke treatments, since end-organ damage dramatically increases data variability and compromises animal survival. It should however be noted that associated diseases similarly reduce the recovery potential of human stroke patients. For the sake of data interpretation, arterial hypertension models should be studied at stages prior to end-organ damage development.

## 4. Insights from Models of Diabetes and Obesity: Utility as Models Turning Beneficial Neurorestorative Effects into Detrimental Ones

Rat and mouse models of type-1 and type-2 diabetes have been established by inbreeding, targeted mutations, or toxin delivery. In ischemic stroke models, species other than rats or mice have not been studied.

Streptozotocin delivery results in near-complete *β*-cell loss resembling type-1 diabetes when repeatedly applied in rats (Table 3) [42]. Fatty streaks are noted in the heart and aorta. When submitted to cerebral thromboembolism, permanent or transient proximal MCAO, infarct volume, brain edema, hemorrhagic transformation, and neurological deficits were increased in streptozotocin-induced type-1 diabetic rats compared to nondiabetic rats [42–44]. Following cerebral thromboembolism, infarct volume was only modestly reduced and brain hemorrhages increased by tissue-plasminogen activator- (tPA-) induced thrombolysis in streptozotocin-induced type-1 diabetes [43]. Insulin treatment restored the efficacy of tPA-induced thrombolysis, reducing infarct volume, brain edema, and hemorrhagic transformation [45]. The streptozotocin model was adapted by applying streptozotocin only once in middle-aged rats [46] or applying streptozotocin in rats receiving high-cholesterol diet (Table 3) [47]. Single streptozotocin injection provokes partial *β*-cell loss, which is associated with peripheral insulin resistance upon high-cholesterol diet exposure [47]. Both models are type-2 diabetes models. Single streptozotocin injection in middle-aged rats resulted in microvascular thrombosis, blood-brain barrier leakage, and cognitive dysfunction [46]. Following cerebral thromboembolism, middle-aged diabetic rats revealed increased sensorimotor deficits and microvascular injury, reduced neurogenesis and oligodendrogenesis, and impaired dendritic and synaptic plasticity compared to nondiabetic controls [46]. Infarct volume was unchanged [46]. Streptozotocin-induced type-1 and type-2 diabetic rats displayed different responses to neurorestorative therapies. In transient proximal MCAO, mesenchymal stem cell delivery improved neurological recovery, increased angiogenesis, and increased white matter remodeling over up to 28 days in streptozotocin-induced type-2 diabetic rats [47], but induced poor neurological recovery accompanied by aberrant angiogenesis, brain hemorrhages, and animal deaths in type-1 diabetic rats [44]. Extracellular matrix degradation was more severe in type-1 than type-2 diabetic rats, resulting in massive neuroinflammation characterized by M1-macrophage infiltrates [44, 47, 48]. Upon mesenchymal stem cell treatment, macrophage infiltrates shifted to an anti-inflammatory M2-phenotype in type-2, but not type-1 diabetic rats [44, 47, 48]. This process was controlled by advanced glycation end-product receptor [48].

The Lep^ob/ob^ mouse is leptin deficient and the Lepr^db/db^ mouse deficient for leptin receptor. Both mice reveal hyperphagia, obesity, hyperinsulinemia, and hyperglycemia, representing models of type-2 diabetes. In transient proximal MCAO or unilateral hypoxia-ischemia induced by permanent carotid occlusion combined with transient 8% hypoxia (Vannucci model), infarct volume, brain edema, extracellular matrix breakdown, and brain neutrophil and macrophage infiltration were increased at 1-3 days in Lep^ob/ob^ and Lepr^db/db^ mice compared to control mice [49–52]. Rosuvastatin decreased infarct volume after transient proximal MCAO in Lep^ob/ob^ but not wild-type mice by reducing intercellular adhesion molecule-1 level and decreasing brain leukocyte entry [51]. The peroxisome proliferator-activated receptor-*γ* activator darglitazone decreased infarct volume after unilateral hypoxia-ischemia (Vannucci model) in Lep^ob/ob^, but not control mice, by downregulating interleukin-1*β* and tumor necrosis factor-*α* [52]. Oligodendrocyte precursor cell proliferation and white matter myelination were compromised after transient distal MCAO in Lepr^db/db^ compared to wild-type mice [53]. White matter compound action potential conduction and sensorimotor performance were impaired [53]. Microglia/macrophage polarization was shifted towards the M1-phenotype [53]. In cell culture, M1 microglia/macrophages suppressed oligodendrocyte precursor cell differentiation under high-glucose conditions [53].

The KK-A^Y^ mouse is an obese, diabetic heterozygous KK mouse expressing spontaneously mutated yellow obese A^Y^ agouti gene (Table 3). After permanent proximal MCAO, low-dose telmisartan decreased infarct area, neurological deficits, and brain tumor necrosis factor-*α* levels at 24 hours in KK-A^Y^, but not in wild-type mice [54]. The peroxisome proliferator-activated receptor-*γ* inhibitor GW9662 reversed these effects [54].

The Zucker diabetic fatty rat (ZDF) exhibits a combination of hyperphagia, obesity, and diabetes resulting from a spontaneous leptin receptor mutation in the otherwise insulin-resistant Zucker rat strain (Table 3). Compared to Zucker lean controls, ZDF reveal significantly elevated CD11b levels on blood neutrophils [55]. Infarct volume, neurological deficits, brain edema, cerebrovascular neutrophil adhesion, and cerebral interleukin-1*β* expression after transient proximal MCAO were increased [55].

The Goto-Kakizaki rat is a nonobese type-2 diabetic rat obtained by breeding Wistar rats with compromised glucose tolerance (Table 3), after transient proximal MCAO hemorrhagic transformation, but not infarct volume, was increased [56].

### 4.1. Lessons from Diabetes and Obesity Models

Diabetes models are Janus-faced conditions, which may turn beneficial treatment effects into detrimental ones, resulting in poor neurological outcome. They are important assays for evaluating risks of treatment failure. In the acute stroke phase, diabetes models can be used for examining exacerbated brain injury, edema, and hemorrhage [42–46, 49–52, 55, 56]. In the postacute phase, they can be used for exploring the risk of maladaptive neuroplasticity and aberrant angiogenesis [44, 47, 48, 53]. An added value is the utility of diabetes models for studying neuroinflammation [44, 47–55], which is augmented by diabetes and obesity. Thus, diabetes models are very suitable for studying anti-inflammatory treatments. Open questions remain how the duration of preexisting diabetes and antidiabetic treatment influences restorative treatment responses. It needs to be shown whether and how fast glucose lowering reestablishes neurorestorative responses.

## 5. Conclusions from Studies on Risk Factor Models

Risk factor models possess important features allowing us to study treatment concepts in ischemic stroke. Useful models have been developed for evaluating ischemic injury associated with risk factor exposure. Useful models have been established for studying impaired angiogenesis and neuroplasticity. Useful models have been presented for spontaneous stroke. Despite these features, risk factor models have so far not been able to identify treatments that subsequently succeeded in clinical trials in the acute neuroprotection field. A downside of current animal models is that several models require genetic manipulations or artificial surgical interventions (i.e., delivery of toxins/drugs) raising questions about their relevance to human stroke. Some models exhibit risk factor manifestations rarely noted in human stroke patients (i.e., excessive hypercholesterolemia, hypertension, or hyperglycemia). Such models should be interpreted with caution. Reproducible models of stenosing large artery atherosclerosis, intracranial large artery atherosclerosis, or spontaneous thromboembolic stroke are largely lacking. The major reason for the lack of spontaneous thromboembolic stroke models is that rodents normally do not exhibit plaque rupture.

How should risk factor models be refined for stroke studies? Animal models should as closely as possible reproduce risk factor states in stroke patients, which requires that the duration, type, and severity of risk factor exposure are mimicked. Stroke typically develops as a consequence of chronic risk factor exposure. Patients mostly exhibit more than one, often several risk factors. Hence, future animal studies should more stringently model long-term risk factor exposure and exposure to combinations of risk factors. From this perspective, animal models of cerebral microangiopathy associated with metabolic syndrome are particularly promising [7, 23]. Attention should be paid to modest risk factor manifestations, such as disturbed glucose tolerance and prediabetes, which are highly prevalent in stroke patients [57]. If possible, treatment studies should involve middle-aged or aged animals [46].

How should risk factor models practically be applied in the evaluation of new therapies? The most widely used approach is an approach, in which treatment concepts identified in young, otherwise healthy, animals are tested in risk factor animals before studies in stroke patients are performed. The main advantage of this bottom-up strategy is the stepwise translation from simple (healthy animal) to more complex (risk factor animal) models. Its disadvantage is that treatment responses may be attenuated by risk factors or comorbidities, which reduces success chances of later clinical trials. In the opposite approach, treatment concepts are first examined in vascular risk factor animals, subsequently evaluated in otherwise healthy animals and then tested in stroke patients. The main advantage of this top-down approach is the known efficacy of treatments in risk factor settings, which increases the chances that treatments will also be acting in the clinical setting. The main message of this overview is that careful risk factor modeling will allow us to increase the validity of animal studies, which should facilitate the translation of new treatments to stroke patients. Without such risk factor modeling, we might end up in another series of clinical translation failures. The principles underlying neuronal plasticity and brain remodeling in the post-acute stroke phase are complex, probably more complex than those controlling acute postischemic neuronal survival and death. We need to develop a thorough understanding how risk factors and comorbidities compromise postacute stroke recovery processes. Such understanding may guide us to the development of new stroke treatments.

## Figures and Tables

**Table 1:** Animal models of hypercholesterolemia used in ischemic stroke studies.

Animal model	Blood cholesterol concentrations	Systemic vascular pathology	Cerebrovascular features
ApoE^−/−^ mouse	Increased 4.3-8 times on regular diet, elevated additional 1.4-2.7 times on high-cholesterol diet [7, 8]	Fatty streaks in aortic wall and extracranial carotid arteries after 6 weeks that are exacerbated by high-cholesterol diet and then associated with lipid deposition in brain arterioles [7, 9]. Upon high-cholesterol diet atherosclerotic plaque formation within 6 months [9]	Infarct volume, blood-brain barrier permeability, and neurological deficits after transient proximal MCAO or transient distal MCAO increased in ApoE^−/−^ mice on high-cholesterol diet [7–10]. Vasorelaxation and VEGF-induced angiogenesis were compromised, resulting in reduced cerebral blood flow [7–11]. Increase of infarct volume and blood-brain barrier permeability mediated by RhoA overactivation and brain neutrophil invasion [7, 8, 10]

LDL receptor^−/−^ mouse	Increased 1.9-2.4 times on regular diet, elevated additional 1.5 times on high-cholesterol diet [12]	Little vascular abnormalities under normal diet [12]. Upon high-cholesterol diet, atherosclerotic plaque formation in aortic wall and coronary arteries after 5-7 months [12]	Thrombotic occlusions of cerebral microvessels and occasional microhemorrhages in LDL receptor^−/−^ mice on high-cholesterol diet at ~6 months [14]. Infarct volume after transient proximal MCAO unaffected by LDL receptor^−/−^ [16]. Platelet deactivation by antiglycoprotein-Ib and antiglycoprotein-VI antibody reduced infarct volume and neurological deficits in 12-month-old LDL receptor^−/−^ and wild-type mice without increasing hemorrhagic transformation [16]. Glycoprotein-IIb/IIIa inhibition had no effect [16]

Human ApoB transgenic (hApoBTg) mouse	Increased 1.3-1.8 times on regular diet, elevated additional 2.2 times on high-cholesterol diet [13]	Little vascular abnormalities under normal diet [13]. Upon high-cholesterol diet, atherosclerotic plaque formation in aortic wall that is more severe in female than male mice after 4 months [13]	Brain capillary density reduced in hApoBTg mice upon high-cholesterol diet after ~6 months, suggestive of spontaneous microvascular occlusions [15]. Microvascular injury after unilateral common carotid artery occlusion not influenced by transgenic ApoB in mice exposed to high-cholesterol diet [15]

High-cholesterol diet in wild-type mouse	Increased 2.2-3.9 times in C57BL6 mouse [11, 13]	Modest fatty streaks in the aorta even after several months of high-cholesterol diet [13]. Metabolic syndrome characterized by obesity, type-2 diabetes, and hypercholesterolemia [23] with lipid deposition in cerebral arterioles [7]	Blood-brain barrier permeability, calpain-1/2 activity, matrix metalloproteinase-2/9 activity, and brain edema after transient proximal MCAO increased in C57BL6 mice on high-cholesterol diet presumably due to RhoA overactivation [7]. Infarct volume and neuronal injury unchanged [7, 11]

High-cholesterol diet in rat	Increased 2.1-4.2 times in Sprague-Dawley rat [26, 27]	Modest fatty streaks in the aorta even after several months of high-cholesterol diet [26, 27]	Infarct volume, blood-brain barrier permeability, brain edema, brain lipid peroxidation, and brain leukocyte infiltration after transient proximal MCAO increased in Sprague-Dawley rats on high-cholesterol diet [26, 27]. Changes prevented by simvastatin and ginkgolide-B [26]

High-cholesterol diet in rabbit	LDL increased 34.7 times [28]	Atheromas of internal carotid and basilar arteries after 5 weeks of high-cholesterol diet [28]	Number of hemorrhagic infarcts reduced, but infarct size increased in rabbits on high-cholesterol diet exposed to focal cerebral ischemia induced by sephadex-G75 polymer [28]. Increased infarct size associated with accumulation of platelet-rich thrombi in infarct foci [28]

Modified from Reference [6].

**Table 2:** Animal models of arterial hypertension used in ischemic stroke studies.

Animal model	Model origin/characteristics	Systemic vascular pathology	Cerebrovascular features
Spontaneously hypertensive rat (SHR)	Inbred rat obtained by crossing Wistar-Kyoto rats with high blood pressure [29, 30]	Apart from impaired endothelial reactivity, no gross vascular abnormalities, specifically no atheromatosis, vascular thrombosis, fibrinoid necrosis, or hyaline degeneration [30]. Cardiac hypertrophy and renal insufficiency with proteinuria developing at the age of 40-50 weeks	In permanent distal MCAO or photothrombosis, NogoA-neutralizing antibody increased sensorimotor recovery and contralesional corticospinal plasticity in SHR and normotensive rats [32]. Only subtle brain changes of neurotrophic factors and their receptors (BDNF, neutrophin-3/4, TrkA/TrkB) noted in SHR compared to normotensive controls [33]. Postischemic neurogenesis and oligodendrogenesis after transient proximal MCAO prematurely attenuated in middle-aged (i.e., 12-month-old) compared to young (3-month-old) SHR [34]

Dahl salt-sensitive rat (DSR)	Inbred rat obtained by mating Sprague-Dawley rats exhibiting severe hypertension upon high-salt diet (8% NaCl) [37]	Fibrinoid necrosis, hyaline degeneration, and hyperplasia of arterioles and small artery walls [37]. Cardiac hypertrophy, interstitial fibrosis, and cardiac failure at ~4-5 months [37]. Glomerular sclerosis with renal insufficiency	In up to 54% of DSR on high-salt diet, fulminant spontaneous brain infarcts associated with massive aortic thickening, neurological deficits, and death noted [37]. Blood pressure, aortic thickening, stroke size, and animal mortality reduced by adenoviral kallikrein delivery [37]

mRenTg mouse	Transgenic mouse overexpressing mouse renin under a liver-specific albumin promoter/enhancer [16]	Hyaline degeneration and hyperplasia of arterioles and small artery walls. Cardiac hypertrophy and proteinuria noted at 3-8 months. 50% of males died between 6 and 8 months of age	After transient proximal MCAO, infarct volume did not differ between mRenTg and wild-type mice [16]. Platelet deactivation by glycoprotein-Ib or glycoprotein-VI inhibition reduced infarct volume in mRenTg and wild-type mice without increasing brain hemorrhages [16]. Glycoprotein-IIb/IIIa inhibition did not influence infarct volume but increased brain hemorrhages more strongly in mRenTg than wild-type mice [16]

hRenAngTg mouse	Transgenic mouse expressing human renin and human angiotensinogen flanked by their regulatory sequences [38, 39]	Hyaline degeneration and hyperplasia of arterioles and small artery walls	When exposed to high-salt diet and treated with an endothelial NO synthase inhibitor, spontaneous hemorrhages noted in brains of hRenAngTg mice [38]. After permanent proximal MCAO, infarct volume and neurological deficits increased in hRenAngTg mice compared to wild-type mice [39]. Effects attributed to neuronal death-promoting actions of angiotensin-II. *Ex vivo*, neuronal injury after oxygen-glucose deprivation more severe in slices obtained from hRenAngTg than wild-type mice [39]. Effect abolished by angiotensin type-1 receptor inhibitor lorsartan [39]

Renovascular hypertensive rat	Renal hypertension induced by renal artery clipping, leaving contralateral kidney intact (“two kidneys, one clip”) or clipping both kidneys (“two kidneys, two clips”) [40, 41]	Fibrinoid necrosis, hyaline degeneration, and hyperplasia of arterioles and small artery walls associated with microaneurysms and thrombotic vascular occlusions. Cardiac hypertrophy noted in ~25-50% of rats	“Two kidneys, one clip” rats exhibiting increased infarct volume after permanent proximal MCAO [41]. In “two kidneys, two clips” [40, 41] and “two kidneys, one clip” [41] rats, spontaneous cortical and subcortical ischemic infarcts, petechial brain hemorrhages of variable size, and dilated brain ventricles detected after 5-12 weeks. Spontaneous stroke not associated with overt neurological deficits

Modified from Reference [6].

**Table 3:** Animal models of diabetes and obesity used in ischemic stroke studies.

Animal model	Model origin/characteristics	Systemic vascular pathology	Cerebrovascular features
Streptozotocin-induced type-1 and type-2 diabetic rat or mouse	Type-1 diabetes induced by repeated streptozotocin injections, resulting in near-complete *β*-cell loss [42–45]. Type-2 diabetes by single streptozotocin injection in middle-aged rats [46] or rats receiving high-cholesterol diet [47] exhibits partial *β*-cell loss	Fatty streaks in aortic wall and coronary arteries. Rats exhibiting more atherosclerosis than mice, BALB/c mice exhibiting more atherosclerosis than C57BL6 mice. Microvascular thrombosis and blood-brain barrier leakage noted in middle-aged rats with streptozotocin-induced type-2 diabetes [46]	Infarct volume, brain edema, and hemorrhagic transformation increased after cerebral thromboembolism, permanent or transient proximal MCAO in streptozotocin-induced type-1 diabetic rats [42–44]. Infarct volume only modestly reduced by tissue-plasminogen activator thrombolysis [43]. Insulin restored the efficacy of tissue-plasminogen activator thrombolysis, reducing infarct volume, brain edema, and hemorrhagic transformation [45]. Increased sensorimotor deficits, reduced neurogenesis/oligodendrogenesis, impaired dendritic/synaptic plasticity, but unchanged infarct volume after thromboembolism in middle-aged type-2 diabetic rats [46]. Mesenchymal stem cell delivery enhanced neurological recovery, angiogenesis, and white matter remodeling after transient proximal MCAO in type-2 diabetic rats [47], but induced poor neurological recovery associated with aberrant angiogenesis, brain hemorrhages, and massive M1-macrophage infiltrates in type-1 diabetic rats [44, 48]

Lep^ob/ob^ mouse/Lepr^db/db^ mouse	Homozygous mice with spontaneous mutations of leptin (Lep^ob/ob^) or leptin receptor (Lepr^db/db^) genes conferring hyperphagia, obesity, hyperinsulinemia, and type-2 diabetes [49–53]	Fatty streaks in aortic wall and coronary arteries	Infarct volume and brain edema after transient proximal MCAO or unilateral hypoxia-ischemia (Vannucci model) increased in Lep^ob/ob^ and Lepr^db/db^ mice due to excessive extracellular matrix breakdown with brain neutrophil and macrophage infiltration [49–52]. Rosuvastatin decreased infarct volume in Lep^ob/ob^, but not wild-type mice via mechanisms involving intercellular adhesion molecule-1 downregulation and prevention of brain leukocyte entry [51]. Peroxisome proliferator-activated receptor-*γ* agonist darglitazone reduced infarct volume in Lep^ob/ob^, but not control mice [52]. Oligodendrocyte precursor cell proliferation, white matter myelination, and neurological recovery compromised after transient distal MCAO in Lepr^db/db^ compared to control mice [53]. Microglia/macrophage polarization shifted towards M1-phenotype [53]
KK-A^Y^ mouse	Heterozygous mouse with spontaneously mutated yellow obese A^Y^ agouti gene [54]. KK mouse without mutation exhibits glucose intolerance and insulin resistance [54]. Homozygous mutation lethal	Vascular changes modest when exposed to regular diet	Infarct volume, neurological deficits, and brain concentrations of tumor necrosis factor-*α* decreased by low-dose telmisartan in KK-A^Y^ mice exposed to permanent proximal MCAO via mechanisms involving peroxisome proliferator-activated receptor-*γ* activation [54]. At the doses administered, telmisartan did not influence blood pressure

Zucker diabetic fatty rat (ZDF)	Inbred rat with spontaneous leptin receptor mutation conferring hyperphagia, obesity, and diabetes in Zucker rat strain that without this mutation is insulin-resistant [55]	Vascular changes modest when exposed to regular diet. In comparison to Zucker lean controls, significantly elevated CD11b on blood neutrophils and elevated soluble intercellular adhesion molecule-1 levels [55]	Infarct volume, brain edema, neurological deficits, and endothelial neutrophil adhesion increased in ZDF compared to Zucker lean controls exposed to transient proximal MCAO [55]. Cerebral inflammatory response, evaluated by interleukin-1*β*, CXC-motif ligand-1, and E-selectin levels, increased [55]

Goto-Kakizaki rat	Inbred nonobese type-2 diabetic Wistar rat obtained by mating high blood glucose rats [56]	If at all very subtle vascular changes	Hemorrhagic transformation, but not infarct volume, increased in Goto-Kakizaki rats compared to nondiabetic Wistar controls exposed to transient proximal MCAO [56]. Cerebrovascular tortuosity index increased, suggestive of vascular architecture disturbances [56]

Modified from Reference [6].
